# Novel Morbillivirus as Putative Cause of Fetal Death and Encephalitis among Swine

**DOI:** 10.3201/eid2707.203971

**Published:** 2021-07

**Authors:** Bailey Arruda, Huigang Shen, Ying Zheng, Ganwu Li

**Affiliations:** Iowa State University, Ames, Iowa, USA

**Keywords:** Morbillivirus, Paramyxoviridae, encephalitis, placentitis, fetal death, swine, porcine, viruses, zoonoses, respiratory infections, enteric infections

## Abstract

Morbilliviruses are highly contagious pathogens. The *Morbillivirus* genus includes measles virus, canine distemper virus (CDV), phocine distemper virus (PDV), peste des petits ruminants virus, rinderpest virus, and feline morbillivirus. We detected a novel porcine morbillivirus (PoMV) as a putative cause of fetal death, encephalitis, and placentitis among swine by using histopathology, metagenomic sequencing, and in situ hybridization. Phylogenetic analyses showed PoMV is most closely related to CDV (62.9% nt identities) and PDV (62.8% nt identities). We observed intranuclear inclusions in neurons and glial cells of swine fetuses with encephalitis. Cellular tropism is similar to other morbilliviruses, and PoMV viral RNA was detected in neurons, respiratory epithelium, and lymphocytes. This study provides fundamental knowledge concerning the pathology, genome composition, transmission, and cellular tropism of a novel pathogen within the genus *Morbillivirus* and opens the door to a new, applicable disease model to drive research forward.

*Paramyxoviridae* encompasses a group of large (300–500 nm in diameter), enveloped, pleomorphic viruses with RNA genomes of 14.6–20.1 kb. The family comprises of 4 subfamilies and 17 genera that contain >70 species and includes global human and animal viral pathogens of concern (*1*). Currently, the genus *Morbillivirus*, in subfamily *Orthomyxovirinae*, contains measles virus (MeV), rinderpest virus (RPV), peste des petits ruminants virus (PPRV), canine distemper virus (CDV), phocine distemper virus (PDV), cetacean morbillivirus (CMV), and feline morbillivirus (FeMV) (*2*,*3*). 

Morbillivirus genomes encode 6 structural proteins in the following order: nucleocapsid (N) protein, phosphoprotein (P), matrix (M) protein, hemagglutinin (H) protein, fusion (F) protein, and large polymerase (L) protein (*2*). Two nonstructural proteins, C and V, are expressed from the P open reading frame and are thought to interfere with the innate immune response in at least a subset of members of the family *Paramyxoviridae* (*4*).

Morbilliviruses cause respiratory and gastrointestinal disease and profound immune suppression (*5*). Morbillivirus host species experience a similar pathogenesis; infection occurs through inhalation, direct contact with body fluids, or fomites or vertical transmission (*6*–*8*). Carnivore morbilliviruses readily invade the central nervous system (CNS), and all morbilliviruses produce intranuclear viral inclusion bodies containing nucleocapsid-like structures (*1*,*9*,*10*).

Paramyxoviruses known to naturally infect swine include porcine rubulavirus, Menangle virus, Nipah virus, and porcine parainfluenza virus (*11*–*16*). Less well-characterized paramyxoviruses associated with central nervous and respiratory disease in pigs also have been reported (*17*–*20*), but none of these viruses are classified in the genus *Morbillivirus*. Using histopathology, metagenomic sequencing, and RNA in situ hybridization (ISH), we identified a novel morbillivirus in swine as the putative cause of an outbreak of reproductive disease characterized by fetal mummification, encephalitis, and placentitis. 

## Materials and Methods

### Clinical Background and Samples

In early 2020, the Iowa State University Veterinary Diagnostic Laboratory (Ames, IA, USA) received 22 porcine fetuses from 6 litters (A–F) that originated from a commercial breeding herd in northern Mexico for routine diagnostic investigation (Table 1). The breeding herd was comprised 2,000 sows and reported reproductive clinical signs characterized by an increased percentage (18% reported) of mummified fetuses and stillbirths. For negative controls, we used fetal tissues from 2 litters from a 3,000-head sow farm in the United States that was experiencing increased mummified fetuses and stillborn fetuses.

**Table 1:** Clinical data and gross pathology in instances of novel porcine morbillivirus among pig litters*

Litter ID	Sow parity	Crown to rump length, cm (condition of fetuses submitted)	Total born	No. mummified fetuses	No. stillbirths
A	6	24 (S)	13	0	4
B	4	24 (N)	12	0	4
C	6	29.5 (S)	NA	NA	NA
D	2	7 (M), 9 (M), 14 (M), 15 (M), 28 (Mod), 26 (Mod)	9	6	0
E	1	7 (M), 7 (M), 9 (M), 12 (M), 14 (M), 14 (M), 15 (M), 15 (M), 15 (M), 26 (Mod)	NA	NA	NA
F	1	16 (M), 23 (Mod), 19 (S)	9	3	0

*M, mummified fetus; Mod, fetus with moderate autolysis; N, neonatal mortality; NA, not available; S, stillborn fetus.

### Pathology

At necropsy, we recorded the condition, including neonatal morality, mummified fetus, moderate autolysis, or stillbirth, and the crown-to-rump length (CRL) of each fetus or piglet in the case record (Appendix). We processed fixed tissues by standard technique, stained tissues by using hematoxylin and eosin, and performed histologic evaluations. We used sections from the paraffin blocks for ISH.

### Porcine Morbillivirus ISH and PCR

At Iowa State University Veterinary Diagnostic Laboratory, we used RNAscope 2.5 HD Reagent Kit (Advanced Cell Diagnostics [ACD] bio-techne, https://acdbio.com) to perform RNA ISH according to the manufacturer’s instructions for formalin-fixed paraffin-embedded samples (Appendix). We prepared fetal thoracic tissue homogenate from each litter, extracted nucleic acids, and performed PCR similar to previously described methods (*21*). We used fetal heart and lung to perform PCRs for porcine circovirus 2 and 3 (PCV2 and PCV3), porcine parovirus 1 (PPV1), and porcine reproductive and respiratory virus (PRRSV). We used kidney tissue for *Leptospira* sp. PCR (Appendix).

We developed a real-time reverse transcription PCR (RT-PCR) specific for PoMV by using the MBLV-900F and MBLV-988R primers and the MBL-959P probe (Appendix Table 2). In addition, we performed a previously described real-time RT-PCR (*22*) to rule out porcine rubulavirus coinfection (Appendix).

**Table 2:** Pairwise identities of predicted gene and gene products of porcine morbillivirus compared with other paramyxoviruses*

Paramyxovirus	N			P			M			F			A (H)			L	
	CS	AA		CS	AA		CS	AA		CS	AA		CS	AA		CS	AA
*Morbillivirus* (*Orthoparamyxovirinae*)																	
CDV	65.8	69.0		61.4	48.3		67.3	77.6		62.5	64.2		52.7	45.4		68.1	75.2
PDV	66.6	70.3		61.3	50.1		68.0	78.8		62.4	62.6		51.5	44.6		67.5	75.8
CMV	62.7	66.4		57.2	43.9		68.4	77.9		64.1	65.5		48.2	39.9		65.3	72.0
PPRV	60.6	64.9		54.9	41.0		65.6	72.5		62.8	62.9		45.6	34.5		64.5	69.7
RPV	62.8	66.0		54.5	39.7		65.2	73.1		61.9	63.1		44.8	32.6		65.3	70.6
MeV	63.1	65.4		54.2	37.9		65.7	74.6		60.9	63.9		45.3	34.5		65.3	70.2
FeMV	56.3	56.3		44.3	26.0		62.4	60.9		50.2	42.2		31.7	15.7		58.1	56.2
*Salemvirus* (*Orthoparamyxovirinae*)																	
Salem virus	50.7	45.0		33.5	21.2		53.8	47.9		45.9	35.5		27.1	11.9		52.6	46.8
*Narmovirus* (*Orthoparamyxovirinae*)																	
TupPV	44.0	32.3		37.3	19.6		51.6	43.5		46.0	33.3		27.1	10.9		54.2	49.5
MosPV	43.7	36.0		36.7	20.9		54.1	48.8		46.0	35.9		30.1	10.9		54.4	49.6
*Jeilongvirus* (*Orthoparamyxovirinae*)																	
Tailam virus	45.4	35.4		38.9	18.4		53.9	46.8		43.5	33.8		30.3	12.6		52.8	48.1
MmlPV	46.5	34.0		39.0	21.5		52.5	46.4		44.3	32.1		31.2	11.9		53.2	47.8
*Henipavirus* (*Orthoparamyxovirinae*)																	
Nipah virus	42.1	30.3		34.4	18.8		52.7	45.2		45.3	32.1		30.8	11.7		51.2	46.2
Bat Paramyxovirus	42.9	33.1		34.6	16.9		52.8	43.3		46.4	32.6		26.8	9.4		51.1	45.0
*Ferlavirus* (*Orthoparamyxovirinae*)																	
FdlPV	41.7	25.7		35.1	13.3		46.1	34.9		42.9	29.0		28.1	10.9		48.2	39.6
*Aquaparramyxovirus* (*Orthoparamyxovirinae*)																	
AsaPV	38.8	23.0		30.4	11.4		45.2	36.7		43.2	30.4		29.1	9.9		47.9	39.0
*Respirovirus* (*Orthoparamyxovirinae*)																	
BpiPV-3	37.1	20.2		31.5	12.8		45.0	35.7		40.9	25.8		30.4	11.7		48.2	37.3
HPIV-1	38.1	20.4		31.1	11.2		45.2	37.1		42.9	26.8		29.8	9.7		47.9	38.7
*Pararubulavirus* (*Rubulavirinae*)																	
Tioman virus	38.5	24.7		33.6	15.3		38.3	23.3		38.5	23.5		30.3	9.6		40.9	29.4
*Orthorubulavirus* (*Rubulavirinae*)																	
SipPV	37.5	23.7		34.5	11.9		37.7	18		38.1	24.1		30.2	10.1		41.1	29.3
PrPV	38.0	23.8		32.8	12.8		37.6	18.6		39.2	28.6		29.9	10.1		40.8	28.5
*Orthoavulavirus* (*Avulvirinae*)																	
NDV	37.0	23.1		33.8	13.2		34.4	20.8		40.5	25.2		28.7	11.5		40.2	27.1
ApPV	37.3	24.5		33.2	ND		33.7	19.6		41.7	26.2		28.2	11.2		39.9	27.2
*Paraavulavirus* (*Avulvirinae*)																	
AviPV-3	38.4	23.7		33.2	17.1		35.5	17.4		38.3	21.7		29.3	13.4		39.5	26.4
*Mataavulavirus* (*Avulvirinae*)																	
AviPV-2	38.8	26.9		35.2	13.9		35.7	20.8		42.0	26.8		27.2	13.6		39.6	26.8
*Synodovirus* (*Metaparamyxovirinae*)																	
WtlPV	34.9	18.2		ND	ND		39.2	23.7		40.8	27.1		27.1	10.6		44.0	34.3
Unassigned to a genus or subfamily																	
WtPV	34.7	15.8		ND	ND		38.4	16.6		36.5	20.2		ND	8.2		41.8	26.3
WhPV	35.7	15.1		ND	ND		35.1	17.4		30.0	8.9		28.6	11.2		40.4	25.1
WpssPV	34.8	17.2		ND	ND		29.5	ND		35.7	19.0		27.3	8.7		42.7	26.7

*AA, amino acid sequence; AsaPV, Atlantic salmon paramyxovirus; APV, Antarctic penguin virus A; AviPV-2, avian avualavirus 2; AviPV-3, avian avualavirus 3; BpiPV-3, bovine parainfluenza virus 3; CDV, canine distemper virus; CMV, cetacean morbillivirus; CS, coding sequence; FdlPV, Fer-de-lance virus; FeMV, feline morbillivirus; HPIV-1, human parainfluenza virus 1; MeV, measles virus; MmlPV, Mount Mabu Lophuromys virus 1; MosPV, Mossman virus; ND, not detected; NDV, Newcastle disease virus (Avian avualavirus 1); PDV, phocine distemper virus; PPRV, peste des petits ruminants virus; PrPV, porcine rubulavirus; RPV, Rinderpest virus; SipPV, simian parainfluenza virus; TupPV, Tupaia paramyxovirus; WhPV, Wenling hoplichthys paramyxovirus; WpssPV, Wenzhou Pacific spadenose shark paramyxovirus; WtlPV, Wenling triplecross lizardfish paramyxovirus; WtPV, Wenling tonguesole paramyxovirus.

### Metagenomics and Bioinformatics Analysis

We extracted total nucleic acid of 2 pooled fetal thoracic tissue samples and prepared sequencing libraries, as described previously (*23*). The first pool consisted of litters A and B; the second pool consisted of litters D and E. We used the MiSeq platform (Illumina, https://www.illumina.com) to sequence the libraries by using the MiSeq 600-Cycle Reagent Kit v3 (Illumina). We preprocessed raw sequencing reads and classified reads by using Kraken version 0.10.5-β (*24*) with the standard database. We used Kaiju version 1.6.2 (*25*) to classify unclassified reads, and used KronaTools-2.6 (*26*) to generate the interactive html charts for hierarchical classification results. We extracted reads of the virus of interest, morbillivirus, from the classification results for de novo assembly by using ABySS version 1.3.9 (*27*), iva version 1.0.8 (*28*), and Spades version 3.11.1 (*29*). We manually refined the resulting contigs, and then curated and elongated contigs by using BLAST (https://blast.ncbi.nlm.nih.gov), SeqMan Pro (https://seqman.software), and integrated genomics viewer for visualization (*30*). We closed the genome gap by conventional RT-PCR with specifically designed primers.

We used ClustalW (http://www.clustal.org) to generate multiplex sequence alignments. We constructed phylogenetic trees based on whole-genome sequences and amino acid sequences of the L protein from aligned sequences by the maximum likelihood model in MEGA version X (https://www.megasoftware.net). We used L protein sequences because paramyxoviruses currently are classified based on the sequence comparison of L protein, the RNA-dependent RNA polymerase. We evaluated the robustness of the phylogenetic tree by bootstrapping using 500 replicates. We used interactive Tree of Life (iTOL, https://itol.embl.de) to display, manipulate, and annotate bases of the whole-genome sequence and L protein amino acid sequence trees (*31*).

### Genome Gap Closure and Whole Genome Sequencing

We used conventional RT-PCR to close the genome gap and confirm the genome sequence assembled from next-generation sequencing (NGS). We used 1 pair of primers to close the genome gap and 14 pairs to confirm the genome sequence (Appendix Table 2). 

## Results

### Gross Pathology and Pathogen Detection 

Among 22 porcine fetuses from the 6 litters (A–F) submitted for diagnostic investigation, CRL length varied from 7 to 29.5 cm (Table 1). We noted the litter identification, sow parity, CRL by individual fetus and piglet, and total number born and number of affected fetuses in each litter as reported by the sow farm (Table 1). Submitted fetuses were 1 neonatal death, in which necropsy revealed aerated lungs; 3 stillbirths, all of which were full-term, fresh-type fetuses but had fetal atelectasis; 14 mummified fetuses, in which in utero death occurred with sufficient time for complete dehydration of tissue; and 4 fetuses with moderate autolysis, in which in utero death occurred without sufficient time for complete dehydration of tissue. Gross evaluation of stillbirths and the single neonatal death was unremarkable. 

To address differential diagnoses, we used quantitative PCR (qPCR) to detect known swine viral and bacterial reproductive pathogens. We did not detect PCV2, PCV3, PRRSV, PPV1, or *Leptospira* sp. by qPCR or quantitative RT-PCR (qRT-PCR) in any litter.

### Metagenomic Sequencing

We pooled samples of fetal thoracic tissue from litters A and B (A–B), both of which had encephalitis noted histologically. We also pooled fetal thoracic tissue from litters D and E (D–E), which had leukocytes in the epicardium noted histologically. We performed NGS on the 2 pooled samples by using the MiSeq platform (Illumina). After using an in-house bioinformatics analysis pipeline, we detected and identified 693 paramyxovirus-like reads in A–B and 118,772 in D–E. No reads of other pathogens were identified. De novo assembly obtained 2 contigs with 4,869 and 10,456 nt from pooled sample A–B and another 2 contigs from pooled sample D–E. The nucleotide sequences of the contigs from A–B and D–E were 100% identical but the contigs assembled from pooled sample D–E were slightly longer, 5,042 and 10,705 nt. Sequence analysis of the 4 contigs suggested the presence of a previously undescribed paramyxovirus of genus *Morbillivirus*. The 2 shortest contigs had <40% nt identity to PDV (GenBank accession no. KC802221) and CDV (GenBank accession no. AF014953) at the 3′ end. The 2 longer contigs had >60% nt identity to PDV and CDV at the 5′ end. We propose this paramyxovirus be named porcine morbillivirus (PoMV).

### Genome Sequence Characterization

A complete genome sequence of PoMV (GenBank accession no. MT511667) was obtained by using RT-PCR to fill the gap between the 2 contigs. We designed 14 pairs of primers according to the obtained genome sequence and sequenced the RT-PCR products again, confirming the accuracy of the whole-genome sequence. The genome size of PoMV is 15,714 bases and has a G+C content of 45.19%. The 3′ leader sequence of the PoMV is 55 nt with 13/20 initial nt being highly conserved among morbilliviruses (Appendix Figure 1). PoMV has a 5′ trailer sequence of 41 nt, similar to other morbilliviruses that have a trailer sequence of 40 or 41 nt (Appendix Figure 1), except for FeMV, which has an unusually long trailer sequence of 400 nt. The last 11 nt of 5′ trailer sequences are conserved in all morbilliviruses.

The genome of PoMV contains 6 genes, 3′-N-P/V/C-M-F-H-L-5′, similar to other morbilliviruses. The pairwise alignment of the predicted gene and gene products in PoMV and other paramyxoviruses showed the highest nucleotide and amino acid identities with members of the genus *Morbillivirus* (Table 2). Nucleotide identities were 56.3%–66.6% for N, 44.3%–61.4% for P, 62.4%–68.4% for M, 50.2%–64.1% for F, 31.7%–52.7% for H, and 58.1%–68.1% for L; amino acid identities were 56.3%–70.3% for N, 26%–50.1% for P, 60.9%–78.8% for M, 42.2%–65.5% for F, 15.7%–45.4% for H, and 56.2%–75.8% for L. PoMV had the highest identities to PDV and CDV and the lowest to FeMV (Table 2).

We noted PoMV included the conserved N terminal motif MA(T/S)L in morbilliviruses containing the sequence MASL in the nucleoprotein (N) (Appendix Figure 2). We identified a leucine-rich motif at aa positions 4–11 and 70–77 in the N protein of PoMV (Appendix Figure 2). We identified 2 initiation codons in the P/V/C gene of PoMV; the first translates P and V and the second translates C. In addition, we identified a UC-rich editing site, ttaaaagggg, in the P/V/C gene of PoMV. We detected a conserved cleavage site, RRQKRF, ≈114 aa residues from the N terminus of the F protein. The F protein of PoMV also contains 9/10 Cys residues and 3 potential N-glycosylation sites.

### Phylogenetic Analyses

**Figure 1:**
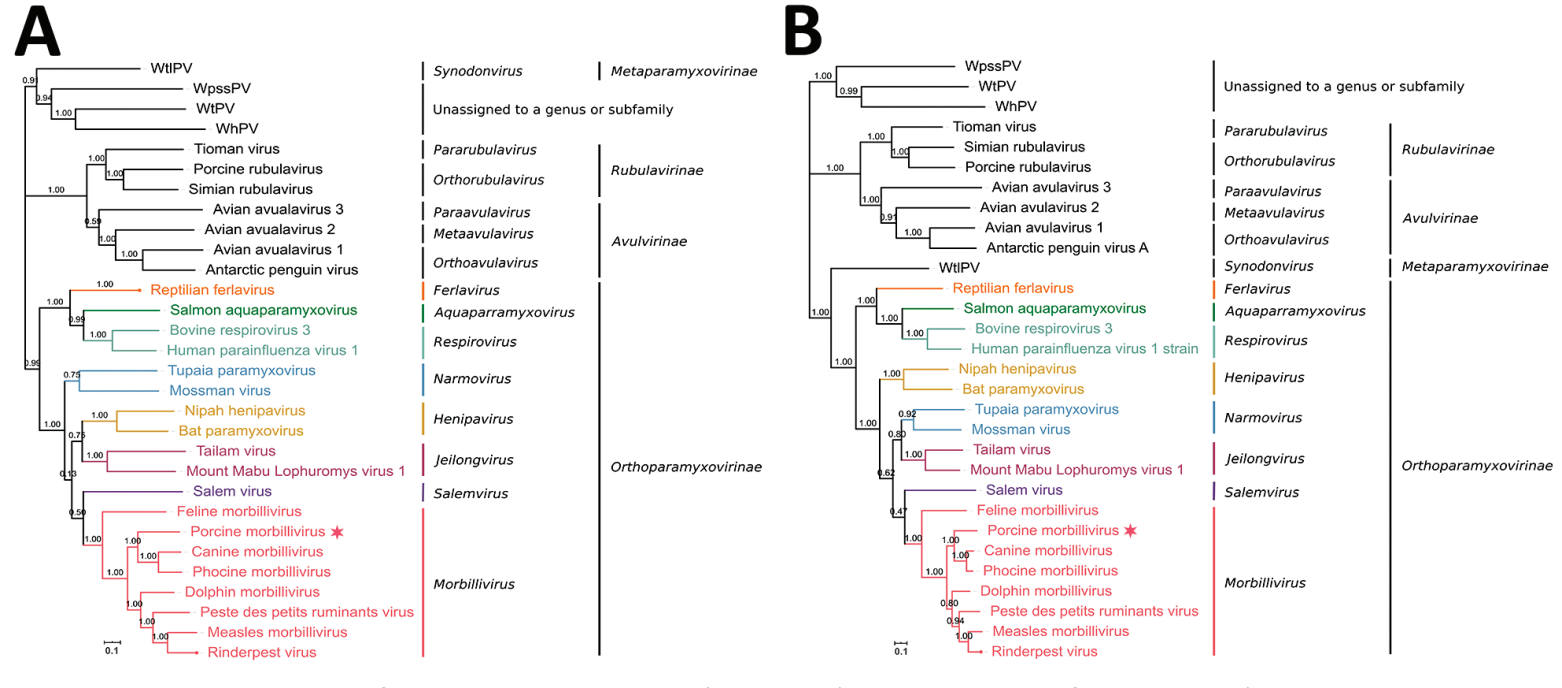
Phylogenetic analysis of novel porcine morbillivirus (PoMV, star) detected among infected swine. A) Phylogenetic analysis of whole genome sequence. B) Phylogenetic analysis of L amino acid sequence. The trees were constructed by maximum likelihood method with bootstrap values calculated from 500 trees and rooted on midpoint. Scale bars indicate nucleotide substitutions per site. WhPV, Wenling hoplichthys paramyxovirus; WpssPV, Wenzhou pacific spadenose shark paramyxovirus; WtlPV, Wenling triplecross lizardfish paramyxovirus; WtPV, Wenling tonguesole paramyxovirus.

We constructed phylogenetic trees by using the whole-genome sequences (Figure 1, panel A) and the predicted aa sequences of the L gene, the RNA-dependent RNA polymerase gene of PoMV and other members of *Paramyxoviridae* (Figure 1, panel B). In both phylogenetic trees, PoMV clustered with other morbilliviruses, with high bootstrap supporting a distinct subgroup (Figure 1). Both phylogenetic analyses also confirmed the findings from the results of pairwise alignment and demonstrated that PoMV was most closely related to CDV and PDV; closely related to CMV, PPRV, MeV, and RPV; and most distantly related to FeMV in the genus *Morbillivirus* (Figure 1). Overall, these data further support that PoMV is a previously undescribed member in the genus *Morbillivirus*, subfamily *Orthoparamyxovirinae*, and family *Paramyxoviridae*.

### Histopathology and PoMV ISH

We performed histologic examination and RNA ISH by litter (Table 3). For all 6 litters, the positive control probe, Ss-PPIB, was positive and the negative control probe, DapB, was negative on the single slide assayed (data not shown). PoMV RNA was not detected by ISH in the cerebrum and cerebellum of 2 stillborn fetuses from unaffected litters used as negative controls (data not shown). 

**Table 3:** Summary of histopathology and RNA in situ hybridization in investigation of novel porcine morbillivirus in pig litters*

Litter ID	Cerebrum			Cerebellum			Lung			Heart			Spleen			Kidney			Liver			Placenta	
	Histo	ISH		Histo	ISH		Histo	ISH		Histo	ISH		Histo	ISH		Histo	ISH		Histo	ISH		Histo	ISH
A	N, M, I	+++		I	+++		I	++		U	Neg		U	+		U	+		Auto	Neg		NA	NA
B	N, M, I, S	+++		U	ND		U	++		U	Neg		U	+		U	Neg		U	Neg		NA	NA
C	U	+		U	ND		U	+		U	Neg		Auto	+++		U	ND		Auto	ND		NA	NA
D	NA	NA		NA	NA		U	++		L	Neg		Auto	+++		U	+		Auto	Neg		L	+
E	NA	NA		NA	NA		U	+++		L	+		NA	NA		U	+		NA	NA		NA	NA
F	U	ND		U	ND		U	++		U	Neg		U	Neg		U	ND		U	ND		L	+++

*Auto, autolysis; Histo, histopathology; I, inclusions; ID, identification; ISH, in situ hybridization; L, leukocytes; M, mineralization; N, necrosis; NA, not available; ND, not done; S, satellitosis; U, unremarkable; +, minimal labeling; ++, moderate labeling; +++, abundant labeling.

**Figure 2:**
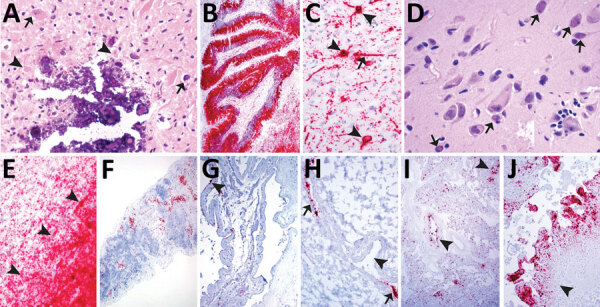
Histologic lesions and porcine morbillivirus (PoMV) RNA in situ hybridization (ISH, red) of tissue of infected swine. A) Histologic section of cerebrum from fetus A stained by hematoxylin and eosin. Arrowheads indicate neuronal necrosis; arrows indicate mineralization and viral inclusion bodies in a neuron and glial cell. B) Cerebellum of fetus A with extensive detection of PoMV by ISH. C) Cerebrum of fetus A; arrowheads indicate ISH labeling within the cytoplasmic and nuclear compartment of neurons; arrow indicates ISH labelling in an axon. D) Cerebrum of fetus B; arrows indicate multiple viral inclusion bodies in neurons; inset displays satellitosis. E) Cerebrum of fetus B showing extensive PoMV detection by ISH. Arrowheads indicate the border of white and gray matter. F) Detection of PoMV by ISH in the spleen of fetus C. G) Detection of PoMV by ISH in a placenta from litter D; arrowhead indicates allantoic epithelium. H) Detection of PoMV by ISH in a renal vessel of a fetus from litter D; arrows indicate the endothelium and arrowhead indicates the vessel lumen. I) Detection of PoMV by ISH in conducting airways (arrowheads) and alveolar septa in the lung of fetus from litter E. J) Detection of PoMV by ISH in the allantoic connective tissue of the placenta and leukocytes from litter F; arrowhead indicates infiltration of leukocytes.

The single stillborn fetus submitted from litter A had multifocal areas of mineralization associated with neuronal necrosis and rarefaction in the cerebrum and brainstem (Figure 2, panel A). Cerebral vessels were occasionally surrounded by lymphocytes. Eosinophilic intranuclear and intracytoplasmic viral inclusion bodies were in neurons (Figure 2, panel A) and glial cells in the cerebrum and internal granular layer of the cerebellum. Rarely, respiratory epithelium lining bronchi and bronchioles contained intranuclear viral inclusion bodies. Histologic evaluation of the heart, spleen, and kidney was diagnostically unremarkable. Moderate autolysis of the liver precluded a thorough histologic evaluation. ISH detected extensive PoMV RNA in the cerebellum in the external granular layer, molecular layer, internal granular layer, and white matter (Figure 2, panel B) and in neurons and axons in the cerebrum (Figure 2, panel C). We also detected PoMV RNA in clusters of respiratory epithelium lining bronchi and bronchioles, scattered lymphocytes and aggregates of lymphocytes in periarteriolar lymphoid sheaths in the spleen, and aggregates of tubular epithelium in rare tubules within the cortex of the kidney. ISH did not detect PoMV RNA in the heart or liver.

The single full-term piglet submitted from litter B had multifocal mineralization and rare satellitosis in the cerebrum (Figure 2, panel D). Adjacent to the lateral ventricle, marked neuropil rarefaction, mineralization, neuronal necrosis, and leukocyte infiltration were visible. Numerous viral inclusion bodies could be seen in neurons (Figure 2, panel D) and glial cells. Histologic evaluation of the cerebellum, lung, heart, spleen, liver, and kidney was diagnostically unremarkable. PoMV was detected by ISH in the gray matter of the cerebrum with extensive labeling; and in the white matter with less but still abundant labeling (Figure 2, panel E). PoMV also was detected by ISH in low to moderate numbers of respiratory epithelium in multifocal bronchi and bronchioles, and individual lymphocytes were noted in the spleen. PoMV RNA was not detected in the heart, kidney, or liver.

Litter C also was represented by a single stillbirth in which histologic evaluation of the cerebrum, cerebellum, lung, heart, and kidney was diagnostically unremarkable. Moderate autolysis of the liver and spleen precluded a thorough histologic evaluation. However, PoMV was detected by ISH in the endothelial cells of a single vessel in the cerebrum, scattered cells within alveolar septa and numerous lymphocytes within the spleen (Figure 2, panel F). PoMV was not detected by ISH in the heart.

Litter D was represented by 3 mummified fetuses and 2 fetuses with moderate autolysis. In a single section of the heart from a fetus with moderate autolysis, we noted mononuclear leukocytes in the epicardium. Lung and kidney of fetuses with moderate autolysis were unremarkable. Autolysis of the spleen and liver from these fetuses and lung, kidney, and heart from mummified fetuses precluded histologic evaluation. Despite severe autolysis and mineralization, leukocytes were observed in the allantoic connective tissue of the placenta. PoMV was detected by ISH in the alveolar septa, bronchi, and bronchioles of the lung of both fetuses with moderate autolysis as well as bronchi and bronchioles of a mummified fetus. PoMV RNA also was detected in scattered lymphocytes in one fetus with moderate autolysis and abundant lymphocytes in the spleen of the other. Mononuclear leukocytes in the allantoic connective tissue and allantoic epithelium of the placenta also contained PoMV RNA (Figure 2, panel G) as did rare renal tubular epithelium and endothelium of a vessel adjacent to the renal pelvis in a mummified fetus (Figure 2, panel H). PoMV was not detected by ISH in the heart or liver of moderately autolyzed fetuses.

Litter E consisted of 9 mummified fetuses and 1 fetus with moderate autolysis. In a single section of 1 heart, the epicardium contained multifocal mononuclear leukocyte aggregates. The lung and kidney were unremarkable in the fetus with moderate autolysis. Autolysis of the heart, lung, and kidney of mummified fetuses precluded histologic evaluation. PoMV was detected by ISH extensively in the conducting airway epithelium and alveolar septa of the lung (Figure 2, panel I), renal tubules, and in rare leukocytes in the epicardium of the fetus with moderate autolysis. ISH was not performed on mummified fetal tissues.

Litter F was represented by a mummified fetus, a fetus with moderate autolysis, and a stillborn fetus. Abundant mononuclear leukocytes were expanding the allantoic connective tissue of the placenta. Histologic evaluation of the cerebrum, cerebellum, spleen, and liver of the stillborn fetus was diagnostically unremarkable. Kidney and lung of the stillborn and moderately autolyzed fetuses were unremarkable. The heart was unremarkable in all fetuses. Autolysis of the mummified fetus precluded evaluation of the lung and kidney. PoMV was detected in the epithelium of conducting airways and alveolar septa of the mummified fetus and the fetus with moderate autolysis. We noted extensive labeling in the allantoic connective tissue and mononuclear leukocytes throughout the placenta (Figure 2, panel J). PoMV was not detected in the lung, heart, or spleen of the stillborn fetus or heart of the fetus with moderate autolysis.

### PoMV Real-Time RT-PCR

Fetal thoracic tissues from litters A, B, D, and E were further subjected to real-time RT-PCR to approximate the viral load by litter. All were positive for PoMV with fetal thoracic tissues from litters A and E having a higher viral load. Litter A had a quantification cycle (C_q_) value of 19.7; the C_q_ for litter E was 19.4. Fetal thoracic tissues from litters B and D had a lower viral load values; B had C_q_ of 23.4 and D had C_q_ of 20.2. PoMV was not detected by real-time RT-PCR in the fetal thoracic tissues of 2 litters of unaffected fetuses selected as negative controls. In addition, porcine rubulavirus was not detected in any sample by RT-PCR.

## Discussion

We report a novel porcine morbillivirus, PoMV, as a cause of fetal death, encephalitis, and placentitis among 6 swine litters. The synchronous use of 3 independent and complementary lines of evidence, pathology, metagenomic sequencing, and in situ hybridization, aided in PoMV discovery. Although several paramyxoviruses have been found, the existence of a naturally occurring morbillivirus in swine previously was unknown. Analyses of predicted nt and aa sequences of 6 genes revealed that PoMV has the highest nucleotide (31.7%–68.1%) and amino acid (26%–75.2%) identities with members in the genus *Morbillivirus* in the N, P, M, F, H, and L genes (Table 2). Phylogenetic analyses based on the whole genome sequence (Figure 1, panel A) and amino acid sequence of the L gene (Figure 1, panel B) further demonstrated that PoMV forms a distinct cluster in morbillivirus and is most closely related to PDV and CDV.

Experimental inoculation of CDV and PPRV in domestic pigs has resulted in infection (*32*,*33*), but no morbillivirus previously has been known to infect swine naturally. Other viruses within the family *Paramyxoviridae* that infect swine include porcine rubulavirus (genus *Orthorubulavirus*, subfamily *Rubulavirunae*), Menangle virus (genus *pararubulavirus*, subfamily *Rubulavirunae*), Nipah virus (genus *Henipavirus*, subfamily *Orthoparamyxovirinae*), and porcine parainfluenza virus 1 (genus *Respirovirus*, subfamily *Orthoparamyxovirinae*). Among these, only porcine rubulavirus and Menangle virus are thought to cause fetal mummification and stillbirths, as observed with PoMV (*34*,*35*). Histologic lesions noted for PoMV are similar to Menangle virus and included encephalitis, viral inclusion bodies, and nonsuppurative myocarditis (*36*).

Similar to our findings with PoMV, MeV has been reported to be transmitted vertically resulting in premature stillbirth, stillbirth, premature birth, neonatal death, or congenital measles (*6*,*37*,*38*). Viral inclusion bodies have been observed in human congenital MeV infection and MeV was detected in placenta and splenic lymphocytes by immunohistochemistry (*39*). Herein, viral inclusion bodies were commonly observed in the cerebrum and cerebellum (Table 3; Figure 2, panels A,D) and rarely in the respiratory epithelium lining conducting airways. ISH demonstrated presence of PoMV in the placenta (Figure 2, panels G, J) and splenic lymphocytes (Figure 2, panel F), and in the cerebrum (Figure 2, panels C, E), cerebellum (Figure 2, panel B), lung (Figure 2, panel I), and to a lesser extent in the kidney (Figure 2, panel H) and heart. Of note, the degree of viral involvement within the monochorionic placenta and effects of MeV on human monozygotic twins was inconsistent; 1 in utero death at 32 weeks gestation and 1 surviving infant with no clinical signs of MeV infection (*39*). This observation, along with the placentation of swine, large litter size, and observations from other swine viral reproductive pathogens (*40*), likely accounts for the variable effects on litters, which were characterized by fetal and piglet death at various stages of gestation and resulted in fetal mummification, in utero death, and stillbirth, along with the variability of ISH staining observed among fetuses and between litters.

Cellular tropism of PoMV determined by ISH aligns with other morbilliviruses, including MeV. A common entry receptor for morbilliviruses is CD150 or signaling lymphocytic activation molecule (SLAM), which is expressed on activated lymphocytes, dendritic cell subsets, and macrophages and cells in the alveolar lumen and lining the alveolar epithelium (*41*–*44*). PoMV RNA was observed in the alveolar septa, lymphocytes of the spleen, and mononuclear leukocytes in the placenta and epicardium, which suggest that PoMV also might use CD150. Morbilliviruses infect epithelia by using nectin-4, which is expressed on the basolateral surface (*45*,*46*). We detected PoMV RNA in the allantoic epithelium of the placenta, epithelium of bronchi and bronchioles, and in rare instances the renal tubular epithelium. In addition, we detected PoMV in the endothelium of a cerebral and renal vessel, and the most extensive staining was in the cerebrum and cerebellum, including neurons. Previous studies have shown that no detectable expression of SLAM was found in human neurons (*47*) and extremely low expression of nectin-4 was detected in central nervous system cells and tissues (*48*). In contrast, CD46 is a widely distributed complement regulatory protein expressed on all nucleated cells with labeling noted in the cerebral endothelium as well as ependymal cells, neurons, and oligodendrocytes (*47*). Accordingly, PoMV also could use CD46 as seen in some MeV strains.

Our study provides essential information about a newly discovered pathogen within the *Morbillivirus* genus, but much is left to learn. The geographic distribution and species susceptibility of PoMV currently is unknown. Virus isolation to facilitate research, in vitro studies evaluating cell entry receptors, and in vivo studies to further elucidate pathogenesis and generate samples of known status for diagnostic assay development and evaluation are needed. Nonetheless, this discovery opens the door to a new and possibly more applicable model of disease to drive research forward (*49*).

Appendix Additional information on novel porcine morbillivirus causing fetal death and encephalitis among piglets.
