# Supplementary material for: Novel Morbillivirus as Putative Cause of Fetal Death and Encephalitis among Swine
Source: Emerg Infect Dis. 2021 Jul;27(7):1858–66. doi: 10.3201/eid2707.203971 (PMC8237871; doi:10.3201/eid2707.203971)
Supplement: Appendix — Additional information on novel porcine morbillivirus causing fetal death and encephalitis among piglets. [file 20-3971-Techapp-s1.pdf]

Article DOI: https://doi.org/10.3201/eid2707.203971

Novel Morbillivirus as Putative Cause of Fetal Death and Encephalitis among Swine

Appendix

Additional Materials and Methods

Pathology

Heart, lung, and kidneys were collected from all 22 swine fetuses from 6 litters (A–F) submitted to the Iowa State University Veterinary Diagnostic Laboratory. We excluded 1 mummified fetus from litter E with a crown-to-rump length (CRL) of 7 cm. We pooled tissue samples by litter and fixed in 10% neutral buffered formalin. Brain, liver, and spleen from stillbirths, spleen and liver from fetuses with moderate autolysis in litter D, and placenta from litters D and F also were fixed in 10% neutral buffered formalin. Fresh fetal thoracic tissue (lung and heart) and kidney were collected and pooled by litter for metagenomic sequencing, quantitative PCR (qPCR) and reverse transcription PCR (qRT-PCR).

Porcine Morbillivirus in situ Hybridization

Paraffin blocks stored at room temperature from each litter originating from Mexico and the blocks containing the cerebrum and cerebellum from 2 stillborn fetuses originating from the second negative control litter originating from a sow farm in the United States as described above were retrieved and 4 µm sections were trimmed and mounted on Superfrost Plus adhesion slides (VWR, https://us.vwr.com). Slides were then dried overnight at room temperature, deparaffinized, treated with RNAscope Hydrogen Peroxide (Advanced Cell Diagnostics [ACD] bio-techne, https://acdbio.com) immersed in the prepared RNAscope 1X Target Retrieval Reagent (ACD bio-techne), rinsed, and treated with RNAscope Protease Plus (ACD bio-techne) as previously described (*1*). Preheated probes were then dispensed on the samples which then were hybridized for 2 h at 40°C in the HybEZ Oven (ACD bio-techne). Advanced Cell Diagnostics (https://acdbio.com) designed and synthesized the RNAscope probe targeting the

RNA of the L (replicase) gene of PoMV, the RNAscope positive control probe Ss-PPIB, and RNAscope negative control probe DapB.

After probe hybridization, we performed 6 rounds of amplification were performed by using RNAscope 2.5 AMP (ACD bio-techne) and processed slides as previously described (*1*). Slides were visualized by a diagnostic pathologist using an Olympus BX43 Brightfield microscope (Olympus Corporation, https://www.olympus-lifescience.com).

PCR

In brief, porcine reproductive and respiratory virus (PRRSV) was detected by using a commercially available TaqMan NA and EU PRRSV Reagents (Applied Biosystems, https://www.thermofisher.com), following manufacturer's instructions. We used primer and probe sequences for detection of porcine circovirus 2 (PCV2), porcine circovirus 3 (PCV3), porcine parovirus 1 (PPV1), and *Leptospira* (Appendix Table 1). We included VetMAX Xeno Internal Positive Control RNA (Life Technologies Corporation, https://www.thermofisher.com) in the master mix to monitor PCR amplification and detection of PCR inhibition. We included 2 positive extraction controls, 1 negative extraction control, and a negative amplification control. We performed RT-qPCR by using the 7500 Fast Real-Time PCR System (Applied Biosystems) with the following cycling conditions: 1 cycle at 50°C for 5 min, 1 cycle at 95°C for 20 min, 40 cycles at 95°C for 3 s, and at 60°C for 30 s. Samples with a quantification cycle (C_q) value <37 for either PRRSV genotype were considered positive. Samples with a C_q value <35 for PPV1, <35 for *Leptospira* spp, <37 for PCV2, and <37 for PCV3 were considered positive.

Porcine morbillivirus (PoMV) primers and probe were synthesized by Integrated DNA Technologies (https://www.idtdna.com). The real-time PCR reaction was performed in a 25 µL PCR mixture that contained 12.5 µL 2× AgPath-ID RT-PCR Buffer (Applied Biosystems), 1 µL 25X RT PCR Enzyme Mix, 1 µL (0.4 µmol) of each of the primers, 0.5 µL (0.2 µmol) of probe, 4 µL nuclease-free water, and 5 µL extracted RNA. The amplification was performed at 48°C for 10 min, 95°C for 10 min, then 40 cycles at 95°C for 15 s and 60°C for 45 s. Samples with a C_q value <40 were considered positive.

Fetal thoracic tissues from 2 litters that originated from a 3,000-head sow farm in the United States experiencing increased mummified fetuses and stillborn fetuses were used for real-time RT-PCR negative controls. The first litter consisted of 2 mummified fetuses with a crown to

rump length (CRL) of 11 and 16 cm. The second litter consisted of 4 stillborn fetuses with CRLs of 26, 27, 28 and 30 cm.

Conventional RT-PCR for Genome Gap Closure and Whole-Genome Sequencing

We performed RT-PCR in 25 µL mixtures containing 12.5 µL 2× PCR buffer, 0.5 µL SuperScript III RT/HiFi Platinum Taq Mix (Thermo Fisher Scientific, https://www.thermofisher.com), 1 µL (0.4 µmol) of each of the primers, 7.5 µL RNA extract, and 2.5 µL DNase/RNase-free water. The cycling conditions were 60 min at 50°C, 2 min at 94°C, then 40 cycles of 30 s at 94°C, 30 s at 55°C, and 2 min at 68°C, and finally extension at 68°C for 5 min. For the genome sequence confirmation, the RT-PCR reaction system and conditions were the same as those for genome gap closing except for an annealing temperature of 48°C instead of 55°C.

Reference

1. Arruda B, Piñeyro P, Derscheid R, Hause B, Byers E, Dion K, et al. PCV3-associated disease in the United States swine herd. Emerg Microbes Infect. 2019;8:684–98. PubMed https://doi.org/10.1080/22221751.2019.1613176

Appendix Table 1. Real-time quantitative PCR primers and probes used in investigation of novel porcine morbillivirus*

Name	Sequence
PCV2 forward primer	5′-GACTGTWGAGACTAAAGGTGGAACTGTA-3′
PCV2 reverse primer	5′-GCTTCTACACCTGGGACAGCA-3′
PCV2 probe	5′-6-FAM/CCCGTTGGAATGGT/MGB-3′
PCV3 forward primer	5′-TGTWCGGGCACACAGCCATA-3′
PCV3 reverse primer	5′-TTTCCGCATAAGGGTCGTCTT-3′
PCV3 probe	5′-VIC/ACCACAAACACTTGGCTC/MGB-3′
PPV1 forward primer	5′-CCAAAAATGCAAACCCCAATA-3′
PPV1 reverse primer	5′-TCTGGCGGTGTTGGAGTTAAG-3′
PPV1 probe	5′-6-FAM/CTTGGAGCCGTGGAGCGAGCC/IA Black FQ-3′
Leptospira spp. forward primer	5′-AAGCATTACCGCTTGTGGTG-3′
Leptospira spp. reverse primer	5′-GAACTCCCATTTCAGCGATT-3′
Leptospira spp. probe	5′-6-FAM/AAAGCCAGGACAAGCGCCG/BHQ1–3′

*PCV2, porcine circovirus 2; PCV3, porcine circovirus 3; PPV1, porcine parovirus 1.

Appendix Table 2. Porcine morbillivirus real-time quantitative reverse transcription PCR primers and probe used in investigation of novel porcine morbillivirus*

No.	Name	Sequence	Use	Reference
1	MBL-1Fa	5′-ACCAAACAAAGTTGGGTAAG-3′	Genome sequence	This study
2	MBLV-900F	5′-GGGCTAGCAAGCTTTATCCTCAC-3′	Genome sequence	This study
3	MBLV-988R	5′-GTTAACTCACCAGCAAACTCATGC-3′	Genome sequence	This study
4	MBL-2000F	5′-ATGAATCACAGACGGCAAC-3′	Genome sequence	This study
5	MBL-2100R	5′-AACATAGTAGTGCTGTATGTCAGATC-3′	Genome sequence	This study
6	MBL-3300R	5′-CATTTGGTGGAATTCCTTGA-3′	Genome sequence	This study
7	MBL-3200F	5′-GATAAAGTCAAGCAGCCTGGA-3′	Genome sequence	This study
8	MBL-4515R	5′-GTCACTGCTTGGCCCTC-3′	Genome sequence	This study
9	MBL-4415F	5′-CCAGGGGCTGTTCAAGATCCTATAAG-3′	Genome gap closure	This study
10	MBL-5462R	5′-GCCGGTCACATTAGGCATAAGTTTTATAAC-3′	Genome gap closure	This study
11	MBL-5400F	5′-TACAAAATTATGGCACCCG-3′	Genome sequence	This study
12	MBL-6600R	5′-CATAAACAGTGTCTGGGTACTCTCT-3′	Genome sequence	This study
13	MBL-6500F	5′-TTACTTACATTCATCGCGACTGAT-3′	Genome sequence	This study
14	MBL-7700R	5′-CTAGTAAGAGATAAGGCAGGTGATATAA-3′	Genome sequence	This study
15	MBL-7600F	5′-CTTATCACATAGCAAATCAGATATATTCCC-3′	Genome sequence	This study
16	MBL-8800R	5′-CACCATATTTTCTCAACCCATACA-3′	Genome sequence	This study
17	MBL-8700F	5′-CATGCAATAGTGTATTATATCTACAATCC-3′	Genome sequence	This study
18	MBL-9900R	5′-TAAGCGAGGGAAAGTGGTTC-3′	Genome sequence	This study
19	MBL-9800F	5′-CTCTGGGATCTGATTGACGG-3′	Genome sequence	This study
20	MBL-11000R	5′-CTTACAGTCTCATAAAGGTCTATGTCATT-3′	Genome sequence	This study
21	MBL-10900F	5′-GTCTCCCAAAATGTCAACTTG-3′	Genome sequence	This study
22	MBL-12100R	5′-CTGTTGACTAGCACGTGCCT-3′	Genome sequence	This study
23	MBL-12000F	5′-TTAGATTGGGCAAGTGATCC-3′	Genome sequence	This study
24	MBL-13200R	5′-TGTTTGGGACTGCCGAAG-3′	Genome sequence	This study
25	MBL-13100F	5′-AACACAGTCCTCCATTTACATGT-3′	Genome sequence	This study
26	MBL-14300R	5′-CTTCTAAATGCATGGATTTCGTA-3′	Genome sequence	This study
27	MBL-14200F	5′-GACGATTTACTATCATTAATGGGATCTAT-3′	Genome sequence	This study
28	MBL-15665R	5′-AAACAAAATTGGCTAAGGGAAC-3′	Genome sequence	This study
2	MBLV-900F	5′-GGGCTAGCAAGCTTTATCCTCAC-3′	PoMV forward primer	This study
3	MBLV-988R	5′-GTTAACTCACCAGCAAACTCATGC-3′	PoMV forward primer	This study
29	MBL-959P	5′-6-FAM-CAGTGCCGGGTACATAGTTTCTATCCCAA-TAMSp-3′	PoMV probe	This study

*Primers MBLV-900F and MBLV-988R were also used in real-time quantitative reverse transcription PCR; primers MBL-4415F and MBL 5462R also were used to close the genome gap.

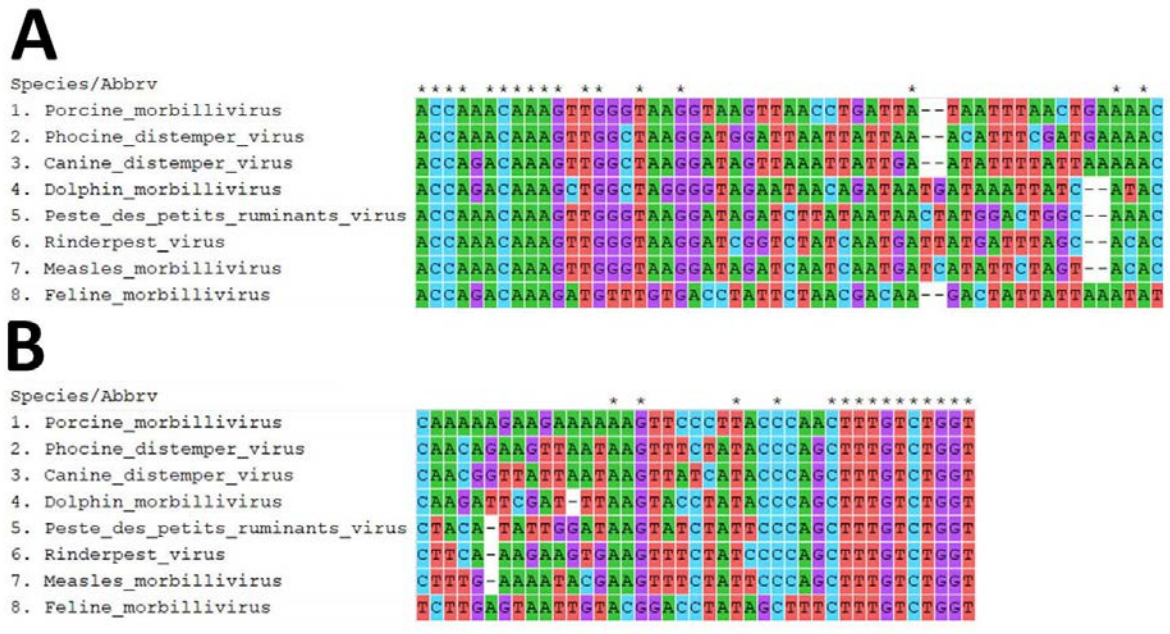

Appendix Figure 1. The 3′ leader and 5′ trailer sequences of porcine morbillivirus (PoMV) and other morbilliviruses. Asterisks indicated conserved nucleotides. A) Alignment of 3′ leader sequences of porcine morbilliviruses. B) Alignment of 5′ trailer sequence of porcine morbilliviruses.

A

Porcine morbillivirus	MASL LKTLSLF KKARDQPPTASGSGGALRGIKHVVIVPIP	40
Phocine distemper virus	.... ..S.... ..T.E...L.......I......I..L..	40
Canine distemper virus	.... ..S.T.. .RT.....L.......I......I..L..	40
Dolphin morbillivirus	..T. .RS.A.. .RNK.RT.LIA.....I......IV..V.	40
Peste des petits ruminants virus	..T. ..S.A.. .RNK.KA.........I....N.I.....	40
Rinderpest virus	.... ..S.A.. .R.K.K..L.A.....I......IV....	40
Measles morbillivirus	..T. .RS.A.. .RNK.K..IT......I.....II.....	40
Feline morbillivirus	.S.. .RS.AA. .RH.E..TAP......IK.L.NTI...V.	40

B

Porcine morbillivirus	GDSTIETRSKLLDRLIKIIGEPDTSGPKQ TGALIGLL SLF	80
Phocine distemper virus	...S.V...R.....VRMV.D.EV....L ..V..SI. ...	80
Canine distemper virus	...S.V...R.....VRLV.D.KIN...L ..I..SI. ...	80
Dolphin morbillivirus	...S.V...R.....VRLA.D.YI....L ..VM.SI. ...	80
Peste des petits ruminants virus	...S.I...R.....VRLA.D..IN.S.L ..VM.SM. ...	80
Rinderpest virus	...S.T...R.....V.MV.D..I....L .....SI. ...	80
Measles morbillivirus	...S.T...R.....VRL..N..V....L ......I. ...	80
Feline morbillivirus	..TV.T...N..F..VY...N...PLSTS ...I.S.. T..	80

Appendix Figure 2. Multiple alignment of N proteins (1–80 amino acid positions) of porcine morbillivirus (PoMV) and other morbilliviruses. The conserved MA(S,T)L motif in morbilliviruses and the 2 leucine-rich motifs at the amino acid positions 4–11 and 70–77 are marked in open boxes. A) The conserved MA(S,T)L motif in morbillivirus. B) The 2 leucine-rich motifs in morbillivirus.
